# Effects of a Rice-Farming Simulation Video Game on Nature Relatedness, Nutritional Status, and Psychological State in Urban-Dwelling Adults During the COVID-19 Pandemic: Randomized Waitlist Controlled Trial

**DOI:** 10.2196/51596

**Published:** 2024-01-22

**Authors:** Seulki Lee, Chisung Yuh, Yu-Bin Shin, Heon-Jeong Lee, Young-Mee Lee, Jungsil Lee, Chul-Hyun Cho

**Affiliations:** 1 Department of Psychiatry, Korea University College of Medicine Seoul Republic of Korea; 2 Department of Medical Education, Korea University College of Medicine Seoul Republic of Korea; 3 Department of Environmental Medicine, College of Medicine, Ewha Womans University Seoul Republic of Korea; 4 Department of Biomedical Informatics, Korea University College of Medicine Seoul Republic of Korea

**Keywords:** video game, digital intervention, nature relatedness, nutritional status, psychological state, COVID-19, urban-dwelling adults

## Abstract

**Background:**

During the COVID-19 pandemic, urban inhabitants faced significant challenges in maintaining connections with nature, adhering to nutritional guidelines, and managing mental well-being.

**Objective:**

Recognizing the urgent need for innovative approaches, this study was designed to explore the potential benefits of a specific digital intervention, the rice-farming simulation game *Sakuna: Of Rice and Ruin*, for nature relatedness, nutritional behaviors, and psychological well-being.

**Methods:**

A total of 66 adults without any prior major psychiatric disorders residing in an urban area were recruited for the study. They were randomly assigned to 2 groups through block randomization: the immediate intervention group (IIG; 34/66, 52%) and the waitlist group (32/66, 48%). Participants in the IIG were instructed to play the game for at least 4 days per week for 3 weeks, with each session lasting from 30 minutes to 3 hours. Assessments were performed at baseline, week 1, and week 3. The Nature Relatedness Scale (NR) and Nutrition Quotient Scale were used to evaluate nature relatedness and nutritional state, respectively. Furthermore, psychological state was assessed using the World Health Organization Quality of Life–Brief Version (WHOQOL-BREF), Brief Fear of Negative Evaluation Scale, Social Avoidance and Distress Scale, Toronto Alexithymia Scale, State-Trait Anxiety Inventory, Center for Epidemiologic Studies Depression Scale Revised, and Korean Resilience Quotient.

**Results:**

This study’s results revealed significant time interactions between the IIG and waitlist group for both the total NR score (*P*=.001) and the score of the *self* subdomain of NR (*P*<.001), indicating an impact of the game on nature relatedness. No group×time interactions were found for the total Nutrition Quotient Scale and subdomain scores, although both groups showed increases from baseline. For psychological state, a significant group×time interaction was observed in the total WHOQOL-BREF score (*P*=.049), suggesting an impact of the game on quality of life. The psychological (*P*=.01), social (*P*=.003), and environmental (*P*=.04) subdomains of the WHOQOL-BREF showed only a significant time effect. Other psychological scales did not display any significant changes (all *P*>.05).

**Conclusions:**

Our findings suggest that the rice-farming game intervention might have positive effects on nature relatedness, nature-friendly dietary behaviors, quality of life, anxiety, depression, interpersonal relationships, and resilience among urban adults during the COVID-19 pandemic. The impact of pronature games in confined urban environments provides valuable evidence of how digital technologies can be used to enhance urban residents’ affinity for nature and psychological well-being. This understanding can be extended in the future to other digital platforms, such as metaverses.

**Trial Registration:**

Clinical Research Information Service (CRIS) KCT0007657; http://tinyurl.com/yck7zxp7

## Introduction

### Background

Urbanization and industrialization have reshaped societies all over the world, with a substantial fraction of populations now residing in city centers [1]. Urban lifestyles, although convenient, may divorce individuals from nature and promote behaviors that can negatively impact both physical and mental health [1,2]. Recognizing the importance of nature for human well-being, it is important for research to be conducted using an integrated approach to examine the effects of urban living on health promotion behaviors, nature relatedness, nutritional status, and psychological states [3-5].

Urban living can engender lifestyle habits that impede connection with nature and hinder balanced nutrition [6]. Many urban dwellers lack access to green spaces, leading to a significant detachment from nature and possibly unhealthy eating habits. The convenience and ubiquity of fast food in urban areas, coupled with limited access to fresh produce, may lead to diets that are high in fat, sugar, and calories. Combined with the sedentary habits required by city living, the lack of connection with nature and poor nutrition escalate the risks of obesity, diabetes, and other health issues [6-8].

The stressors of urban living, social isolation, and alienation from nature can have severe psychological consequences. Restricted access to green spaces contributes to feelings of estrangement from nature, exacerbating stress, anxiety, and depression and undermining the overall well-being. Indeed, studies have shown that exposure to nature can enhance feelings of well-being and mitigate stress [9,10].

The COVID-19 pandemic and the ensuing social distancing policies amplified urban dwellers’ physical and social isolation, increased negative dietary patterns, decreased outdoor physical activities, and escalated psychological distress [11-13]. A rapid rise in obesity and metabolic diseases were associated with such changes in dietary patterns in previously healthy young adults in South Korea during the COVID-19 pandemic [14]. Moreover, mental health, which is highly correlated with obesity and metabolic health, became a serious issue during the pandemic for similar reasons, such as individual isolation measures in closed spaces and detachment from the surrounding community and nature [14]. Because future pandemics are a real possibility, urban dwellers’ susceptibility necessitates the development of strategies to promote their physical and psychological well-being [15,16]. These efforts are not only important for pandemic preparedness but also vital interventions in the modern, urban-centric society.

The video game industry, which saw an expansion during the era of social distancing, presents an opportunity to address urban dwellers’ disconnection from nature and associated health issues [17]. The World Health Organization has suggested that video games may have positive effects for alleviating stress and social isolation [18]. Recently, games and game-like software have been deployed in clinical contexts for health promotion and disease treatment, as well as to promote psychological well-being, highlighting their potential as digital therapeutics [19].

Gamification has become increasingly prevalent in health interventions, capitalizing on the persuasive capabilities of technology to effectuate positive behavior changes [20]. By implementing game mechanics, including points and levels, in nongame contexts, this strategy increases participation and encourages behavior modification [21]. A substantial proportion of the examined studies yielded inconclusive results, indicating that although gamification may be beneficial, its effects are not universally favorable and may differ depending on the context and quality of implementation. Although the literature frequently discusses short-term engagement, the results regarding long-term impact and intrinsic motivation are inconsistent, necessitating further investigation into the ways in which extrinsic motivators can evolve into sustained intrinsic motivation [21].

*Sakuna: Of Rice and Ruin*, a rice-farming action role-playing game, provides a unique blend of entertainment and cultural education [22]. Players assume the role of Sakuna, a harvest goddess who has been banished to an island where she must battle demons and cultivate rice. The game introduces players to the intricacies of traditional rice farming and ties these agricultural practices directly to the character’s progression. We believe that this immersive experience could enhance players’ connection with nature, even in a game-based setting, and provide psychological benefits through exposure to slow and methodical tasks.

As mentioned earlier, healthier food choices, such as traditional homemade rice-based meals in South Korea, could lead to a decrease in obesity and metabolic and mental health issues [14]. Rice is the staple food for more than half of the world’s population, and its cultivation plays an important role in Asian culture. In South Korea and Japan, rice is deeply associated with cultural identity, memories of family, and the community [23]. Healthy homemade meals are mostly perceived to be rice-based meals, which tend to be more balanced, higher in nutrients, and lower in calories than Western foods; several studies have been published on the association of rice-centered Japanese diet with the quality of life, sleep, impulsivity, depressive symptoms, and biochemical changes [24,25].

### This Study

Therefore, we hypothesized that engaging in this rice-farming game would lead to a positive perception of nature and a rice-based balanced diet, which could subsequently affect actual food choices. Improved nature relatedness could positively influence psychological well-being and mood, which, in turn, could also help in choosing a healthier diet in real life. This study aims to explore the potential impacts of playing *Sakuna: Of Rice and Ruin* on nature relatedness, nutritional status, and psychological well-being among urban dwellers during the COVID-19 pandemic, especially during the period of social distancing. We investigated the game’s ability to provide a meaningful and accessible connection with nature and improve nutritional behavior and its potential influence on players’ mental health, focusing on the game’s realism and immersive content.

## Methods

### Participants

Our study used a recruitment strategy involving web-based bulletin boards of social network services and gaming communities in South Korea. The web-based recruitment process included posting institutional review board–approved announcements on our laboratory’s official website and sharing the link to this page on YouTube (Google LLC), Naver (Naver Corp), Twitch (Amazon, Inc), and 8 other game-related web-based platforms. From those who applied, we included only young adults aged 19 to 40 years with a minimum residency period of 6 months in the urban area at the time of study enrollment. The exclusion criteria included individuals diagnosed with major psychiatric disorders, game addiction, mental retardation, traumatic brain injury, or epilepsy or those who had used the game *Sakuna: Of Rice and Ruin* within the last 6 months.

Once potential participants were identified, they were sent a brief video explaining research consent requirements via email. Subsequently, an electronic signature of the web-based informed consent form was obtained from all participants. Of the 71 individuals screened, 68 (96%) agreed to participate and provided their informed consent. We used block randomization to assign participants equally to the immediate intervention group (IIG) and waitlist group (WLG), each of which initially included 34 members. However, 2 (6%) out of 34 individuals from the WLG could not be contacted before the start of the study. Therefore, a final total of 32 participants were in the WLG, resulting in 66 participants overall.

The study required participants to engage in the *Sakuna: Of Rice and Ruin* game under specific conditions. Participants were asked to use the game’s PC version if possible, although the use of the Nintendo Switch (Nintendo Co, Ltd) and PlayStation (Sony Interactive Entertainment) versions was also permitted. The game had a total running time of 35 hours, and participants were expected to play the game for at least 4 days per week for 3 weeks, with each session lasting from 30 minutes to 3 hours in length. We set a maximum daily playtime of 5 hours to prevent potential game addiction, with a strongly recommended 10-minute break time. An additional week was granted to any participant who was unable to complete the game within the initial 3 weeks.

Participants were required to purchase the game independently for the purpose of the study, thus ensuring that each held individual copyright. The researchers did not provide the game to the participants. In addition, the study design involved real-time remote monitoring of gaming activities. The research team observed participants’ in-game progress through instant messaging platforms, where participants were instructed to share snapshots of the game’s start, pause, and finish screens as well as any achievements obtained, to track their engagement with the game.

**Figure 1:**
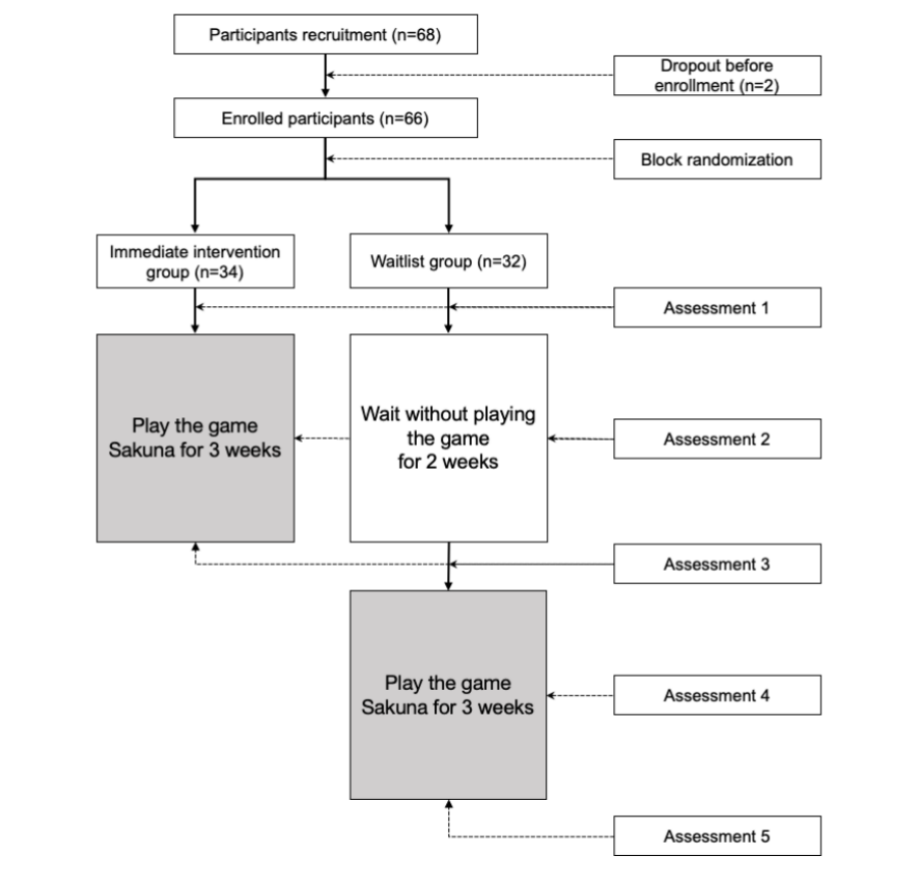
Schematic research flow. Comparison of the waitlist group and immediate intervention group (white), and game intervention in the entire sample (gray).

For the IIG, the gaming intervention took place in April 2021; the first assessment occurred before the game-playing phase, and subsequent evaluations occurred at the end of weeks 1 and 3. The WLG continued their usual daily activities for the first 2 weeks, starting the game only after this period. They also underwent assessments before the gaming phase and at the end of weeks 1 and 3. All the assessments were conducted via a web-based survey site. Figure 1 presents the schematic research flow with evaluations and procedures.

### A Rice-Farming Video Game Digital Intervention (Sakuna: Of Rice and Ruin)

#### Overview

**Figure 2:**
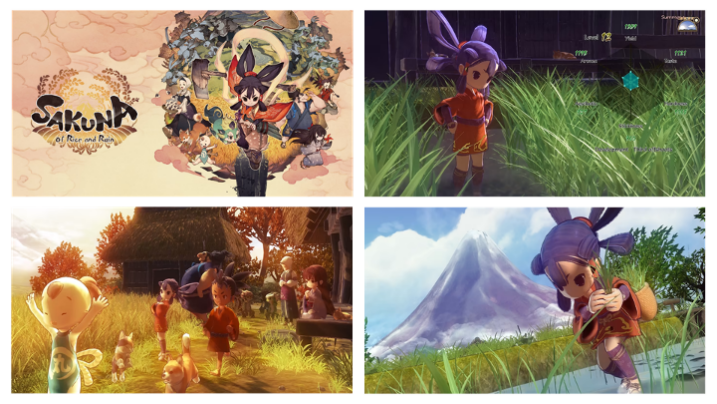
Key scenes from the Sakuna: Of Rice and Ruin game (images used with permission from Edelweiss, the video game developers).

*Sakuna: Of Rice and Ruin* (Marvelous Inc) is an action role-playing simulation game that provides a distinctive blend of agriculture and action gameplay, thus offering a unique gaming experience. The game is divided into 2 primary sections: an agricultural simulator, in which players must cultivate rice, and an action segment, in which players must combat demonic creatures using farming tools (Figure 2). The agrarian component spans several in-game seasons and involves multiple stages. These multiple stages range from tilling the soil and transplanting rice to adding fertilizer to managing water levels to weeding to eventually harvesting and threshing rice (Multimedia Appendices 1 and 2).

Each successful rice harvest permanently enhances the character’s abilities. The quality of the harvest can be improved through meticulous crop management. Players who complete each task access new skills that expedite and enhance the cultivation process. The action element of the game features combat against demonic animals, for which rice harvests bolster players’ strength. Set in nature and modeled around real-world farming practices, it is reasonable to assume that the game may be influenced by the user’s connection with nature, nutritional status, and psychosocial state.

In addition to rice farming, the game emphasizes the importance of traditional family dinners and a well-balanced diet. Sakuna, with her human friends, such as family, makes her own healthy daily dinner from the available natural ingredients (gathered, hunted, harvested, or stored), which are relatively low in calories but rich in nutrients. The available sources for the menu include various grains, 10 types of meat, 11 types of seafood and freshwater fish, 18 types of vegetables and fruits, and 15 types of natural sauce and plants. Sharing meals with family and having dinner conversations in the virtual world could make the player feel at *home* in the game. When Sakuna becomes picky about food, her friends encourage her to try a balanced meal. The player should consider a realistic and sustainable meal choice, as there are expiration periods for the natural food ingredients; expired foods cannot be consumed and should be turned into fertilizers. Furthermore, the menu choice is directly linked to Sakuna’s power and ability to explore the next morning. Eating a well-balanced meal increases Sakuna’s natural healing potential; missing a meal or major nutrients reduces the healing potential to zero, making it difficult to further explore or play an action. A summary of the game components is presented in Multimedia Appendix 3.

The game and its associated elements may contribute to the dietary effects outlined in Textbox 1.

Dietary outcomes might also be shaped by the profound psychological impacts of the game. The major hypothesis of this study was that as the participants were immersed in a virtual green environment with natural background music, their nature relatedness would improve, which could lead to reduced stress, improved mood and resilience, and enhanced mindfulness. These positive psychological effects could subsequently improve the participants’ dietary choices, such as by bringing about a reduction in stress eating (thus reducing the consumption of fast foods) and promoting mindful eating. In addition, increased nature relatedness might lead participants to feel a connection with food sources and to place greater value on them, with shifts toward natural food preferences and proenvironmental behaviors, such as sustainable food choices. However, these could not be fully determined by this study alone; further research is necessary to determine a clearer association between game elements and their effects.

Using *Sakuna: Of Rice and Ruin*, we evaluated our study participants’ affinity to nature, mental health, and nutritional eating patterns. In particular, we focused on their enhanced awareness of rice cultivation and rural areas, their mental health, and their health behaviors in a context that required the application of their knowledge of rice farming.

**Dietary effects of the game.**

- Increased appreciation for a well-balanced diet (with grains, plants, and good protein sources): with an emphasis on balanced nutrition contributing to the character’s power and natural healing potential, participants may develop a positive perception of well-balanced homemade food and wholesome nutrition with minimal processing. Experiencing the process of rice farming may deepen players’ understanding and appreciation of the labor and resources required to produce rice and whole grains. In the postgame interview, some of the participants responded that after experiencing the milling process, they realized how precious the grains were and even
  purchased
  the specific types of whole grains in their real life.
- Shift toward natural or homemade food and reduced fast food consumption: the simulation of traditional home cooking and eating natural foods in the game could influence the player to shift toward natural food selection or homemade meals rather than processed or fast foods. Some participants mentioned that after playing this game, they felt more fun in cooking and tried home cooking more frequently, which reduced the consumption of outside foods. A participant said, “I tried cooking at home the traditional dish I had in Sakuna because it looked so good.”
- Sociocultural reinforcement of healthy food choices: Through daily interaction with nonplayer characters (friends at family dinners and the community), participants are reinforced of the importance of well-balanced diets and traditional Japanese-style homemade meals. Although the sociocultural effect significantly influences Sakuna’s dietary practice in the virtual world, it could also influence participants’ dietary practices in real life.
- Sustainable dietary behavior: Owing to the expiration periods of natural ingredients and foods, participants should make a realistic choice of ingredients, considering both their freshness and nutritional values. Repeatedly experiencing the process of creating natural fertilizers from remnant foods can influence participants into making their dietary behaviors sustainable.

#### Assessment of Nature Relatedness and Nutritional Status

The Nature Relatedness Scale (NR) and Nutrition Quotient Scale (NQ) were used to assess nature relatedness and nutritional status, respectively. The NR measures and evaluates an individual’s connection with nature, their worldview with respect to nature, and their physical familiarity with nature across 3 subscales: *self*, *perspective*, and *experience* [26]. The reliability coefficients for the NR in the baseline and end evaluations were 0.693 and 0.569, respectively. The NQ measures and evaluates the quality and nutritional value of individuals’ meals. It assesses several aspects of a person’s dietary habits across 4 subscales: *balance*, *diversity*, *moderation*, and *dietary behavior* [27]. Balance is the frequency of intake of fruits, eggs, dairy products, beans or bean products, fish, and breakfast. Diversity indicates the level of deviated food habits and the frequency of vegetable and water intake. Moderation is the frequency of having sweet and greasy bread, processed beverages, instant noodles, sugar-sweetened beverages, late-night snacks, and street foods. Dietary behavior includes checking nutrition labels, performing exercises for more than 30 minutes, and washing hands before meals.

#### Assessment of Psychological State

We used multiple scales and inventories to evaluate the psychological states of participants (Textbox 2).

**Instruments used to assess the psychological states of participants.**

**The World Health Organization’s Quality of Life–Brief Version (WHOQOL-BREF)**

- The overall quality of life and well-being were assessed across 4 subscales: physical health, psychological health, social relations, and environment [28]. The reliability coefficients for the WHOQOL-BREF scale in the baseline and end evaluations were 0.895 and 0.925, respectively.

**The Brief Fear of Negative Evaluation Scale (BFNE)**

- This scale measures the fear of being negatively judged by others, where higher scores indicate greater fear [29]. The reliability coefficients for the BFNE scale in the baseline and end evaluations were 0.725 and 0.659, respectively.

**The Social Avoidance and Distress Scale (SADS)**

- This self-report tool assesses social anxiety and the tendency to avoid uncomfortable social situations [30]. The reliability coefficients for the SADS in the baseline and end evaluations were 0.940 and 0.958, respectively.

**The Toronto Alexithymia Scale (TAS)**

- This self-report measure assesses alexithymia, a personality trait characterized by difficulties in recognizing and expressing emotions [31]. The reliability coefficients for the TAS in the baseline and end evaluations were 0.940 and 0.884, respectively.

**The State-Trait Anxiety Inventory (STAI)**

- This inventory measures state and trait anxiety in both research and clinical practice contexts [32]. The reliability coefficients for the STAI–State in the baseline and end evaluations were 0.933 and 0.950, respectively. The reliability coefficients for the STAI–Trait in the baseline and end evaluations were 0.938 and 0.950, respectively.

**The Center for Epidemiologic Studies Depression Scale Revised (CESD-R)**

- This scale comprises 20 questions about various symptoms of depression as they may have appeared over the previous week [33]. The reliability coefficients for the CESD-R in the baseline and end evaluations were 0.915 and 0.912, respectively.

**The Korean Resilience Quotient (KRQ)**

- This measure assesses resilience across 3 areas: self-regulation ability, interpersonal relationships, and positivity [34]. The reliability coefficients for the KRQ scale in the baseline and end evaluations were 0.946 and 0.959, respectively.

**The General Self-Efficacy Scale (GSE)**

- This scale measures individuals’ beliefs in their own abilities and assesses their confidence across various problem situations [35]. The reliability coefficients for the GSE in the baseline and end evaluations were 0.888 and 0.918, respectively.

**The Spiritual Well-Being Scale (SWBS)**

- This self-report measure assesses spiritual well-being, which refers to individuals’ sense of purpose, connection to something greater than themselves, and inner peace [36]. The reliability coefficients for the SWBS in the baseline and end evaluations were 0.895 and 0.905, respectively.

### Statistical Analyses

Data analysis was performed using SPSS (version 24, IMB Corp), incorporating a range of statistical tests, such as 2-tailed *t* tests, chi-square tests, and repeated-measures ANOVA. Repeated-measures ANOVA was specifically used to compare 2 groups of repeated observations captured at baseline, after 1 week of gameplay, and after 3 weeks of gameplay in the IIG and at the corresponding time points in the WLG. Given that the digital game intervention was eventually implemented in both groups, the 2 groups were amalgamated into a single sample (sample of the actual same gameplay at each time point in the IIG arm and after the wait in the WLG arm) to analyze intervention-induced changes. A *P* value of less than .05 was established as the threshold for statistical significance in all analyses. As a total of 10 psychological scales were applied to the analysis, multiple correction was performed. Bonferroni correction was performed for 7 scales (World Health Organization Quality of Life–Brief Version [WHOQOL-BREF], Brief Fear of Negative Evaluation Scale, Social Avoidance and Distress Scale, Toronto Alexithymia Scale, State-Trait Anxiety Inventory [STAI]–State, STAI–Trait, and Center for Epidemiologic Studies Depression Scale Revised) that were measured twice during the study period and for 3 scales (Korean Resilience Quotient, General Self-Efficacy Scale, and Spiritual Well-Being Scale) that were measured thrice during the study period.

### Ethical Considerations

This study underwent a thorough ethical review and was approved by the Chungnam National University Sejong Hospital Institutional Review Board (reference 2021-01-012) and the Clinical Research Information Service (CRIS; reference KCT0007657). Written informed consent was obtained from all parents of the participating children, and informed consent was obtained from all participating children. The study data were anonymized and unidentified. Participants received compensation of KRW ₩70,000 (US $50) for their involvement in the study.

## Results

### Overview

Table 1 presents the characteristics of all 66 participants. There were no significant differences between the IIG (n=34) and the WLG (n=32) in terms of age, sex, socioeconomic status, residence type, or marital status (all *P*>.05).

**Table 1:** Comparison of participant characteristics.

Characteristics			Total (n=66)		Groups					Statistics				*P* values	
					IIG^a^ (n=34)		WLG^b^ (n=32)								
Age (y), mean (SD)			27 (5.07)		28 (5.04)		27 (5.10)		*t*_64_=0.7				.98		
**Age group (y), n (%)**											*χ*^2^_1_=0.4				.53
	19-29	45 (68)		22 (65)		23 (72)									
	30-40	21 (32)		12 (35)		9 (28)									
**Sex, n (%)**											*χ*^2^_1_=0.01				.98
	Male	56 (85)		29 (85)		27 (84)									
	Female	10 (15)		5 (15)		5 (16)									
**Socioeconomic status, n (%)**											*χ*^2^_3_=6.6				.09
	Wealthy	26 (39)		12 (35)		14 (44)									
	Moderate	26 (39)		18 (53)		8 (25)									
	Temporary	11 (17)		3 (9)		8 (25)									
	Poor	3 (5)		1 (3)		2 (6)									
**Residence type, n (%)**											*χ*^2^_2_=1.4				.50
	Living alone	15 (23)		9 (27)		6 (19)									
	Living with another person	10 (15)		6 (18)		4 (12)									
	Living with ≥2 persons	40 (61)		18 (55)		22 (69)									
**Marital status, n (%)**											*χ*^2^_1_=0.7				.79
	Unmarried	57 (86)		29 (85)		28 (87)									
	Married	9 (14)		5 (15)		4 (13)									

^a^IIG: immediate intervention group.

^b^WLG: waitlist group.

### Impacts of the Game on Nature Relatedness and Nutritional Status in the IIG and WLG

Analysis of the impact of the game on nature relatedness and nutritional status (as shown in Table 2 and Multimedia Appendix 4) revealed significant group×time interactions for both the total NR score (*F*_1,60_=13.418; *P*=.001) and the *self* subscale of the NR (*F*_1,62_=16.977; *P*<.001). The total NQ score (*F*_2,116_=6.209; *P*=.003) and moderation (*F*_2,118_=3.954; *P*=.02) and dietary behavior (*F*_2,124_=12.619; *P*<.001) subscale scores generally increased from baseline in both groups, but no group×time interactions were found.

**Table 2:** Comparison of the impact of the game on nature relatedness and nutritional status between the immediate invention group (IIG) and waitlist group (WLG).

Measures and group				Baseline, mean (SD)		1 week after starting the game or 1 week after waiting for the game, mean (SD)		3 weeks after starting the game or 2 weeks after waiting for the game, mean (SD)		Group×time interaction, *F* test (*df*)				*P* values				Time effect, *F* test (*df*)				*P* values		
**NR^a^**																								
	**Total NR**										13.418 (1, 60)				.001				10.053 (1, 60)				.002	
		IIG	56.59 (6.95)		N/A^b^		61.22 (6.00)																	
		WLG	58.07 (7.25)		N/A		57.73 (7.31)																	
	**Self**										16.977 (1, 62)				<.001				18.185 (1, 62)				<.001	
		IIG	24.74 (6.36)		N/A		28.62 (5.51)																	
		WLG	26.07 (5.15)		N/A		26.13 (5.48)																	
	**Perspective**										0.297 (1, 63)				.59				0.051 (1, 63)				.82	
		IIG	15.45 (3.77)		N/A		15.55 (3.36)																	
		WLG	16.56 (3.00)		N/A		16.34 (3.13)																	
	**Experience**										2.453 (1, 63)				.12				0.682 (1, 63)				.41	
		IIG	15.79 (2.47)		N/A		16.39 (2.05)																	
		WLG	15.56 (2.82)		N/A		15.38 (2.37)																	
**NQ^c^**																								
	**Total NQ**										1.461 (2, 116)				.24				82.038 (2, 116)				.003	
		IIG	48.00 (11.59)		49.78 (10.62)		51.46 (11.50)																	
		WLG	50.15 (10.65)		51.07 (8.50)		51.36 (9.14)																	
	**Balance**										0.486 (2, 126)				.62				1.798 (2, 126)				.17	
		IIG	28.64 (15.53)		28.85 (14.24)		29.81 (15.43)																	
		WLG	31.92 (14.92)		30.68 (13.24)		33.81 (17.77)																	
	**Diversity**										1.319 (2, 126)				.27				1.670 (2, 126)				.19	
		IIG	46.75 (19.36)		50.58 (18.88)		51.29 (16.86)																	
		WLG	49.60 (15.97)		50.57 (13.58)		49.45 (13.58)																	
	**Moderation**										0.950 (2, 118)				.39				3.954 (2, 118)				.02	
		IIG	69.65 (12.18)		69.80 (12.35)		73.54 (12.97)																	
		WLG	68.31 (11.93)		69.06 (11.28)		70.01 (13.69)																	
	**Dietary behavior**										1.733 (2, 124)				.18				12.619 (2, 124)				<.001	
		IIG	40.91 (17.46)		45.68 (13.84)		47.76 (16.60)																	
		WLG	44.91 (19.78)		48.24 (17.91)		47.91 (19.52)																	

^a^NR: Nature Relatedness Scale.

^b^N/A: not applicable.

^c^NQ: Nutrition Quotient Scale.

### Impact of the Game on Psychological State in the IIG and WLG

Analysis of the impact of the game on psychological state with Bonferroni correction (Table 3 and Multimedia Appendix 4) showed a significant group×time interaction concerning the total WHOQOL-BREF score (*F*_1,62_=7.652; *P*=.049). Scores for the psychological (*F*_1,64_=6.530; *P*=.01), social (*F*_1,63_=9.677; *P*=.003), and environmental (*F*_1,63_=4.250; *P*=.04) subscales showed a significant time effect at week 3 compared with baseline, but no significant group×time interaction was observed. No significant time effects or group×time interactions were found for the other psychological scales (all *P*>.05).

**Table 3:** Comparison of the impact of the game on psychological state between the immediate intervention group (IIG) and waitlist group (WLG).

Measures and group				Baseline, mean (SD)		1 week after starting the game or 1 week after waiting for the game, mean (SD)		3 weeks after starting the game or 2 weeks after waiting for the game, mean (SD)		Group×time interaction, *F* test (*df*)			*P* values			Time effect, *F* test (*df*)			*P* values	
**WHOQOL-BREF^a^**																				
	**Total score**									7.652 (1, 62)			.049^c^			8.862 (1, 62)			.02^c^	
		IIG	87.79 (11.84)		N/A^b^		93.06 (13.08)													
		WLG	89.65 (12.90)		N/A		89.84 (12.30)													
	**Physical**									3.649 (1, 63)			.06			1.433 (1, 63)			.24	
		IIG	24.29 (3.98)		N/A		25.56 (4.51)													
		WLG	24.65 (5.05)		N/A		24.35 (4.37)													
	**Psychological**									3.192 (1, 64)			.08			6.530 (1, 64)			.01	
		IIG	19.44 (3.82)		N/A		20.68 (4.25)													
		WLG	19.56 (4.19)		N/A		19.78 (3.78)													
	**Social**									3.140 (1, 63)			.08			9.677 (1, 63)			.003	
		IIG	9.68 (1.97)		N/A		10.62 (1.81)													
		WLG	9.61 (2.19)		N/A		9.87 (2.16)													
	**Environment**									3.877 (1, 63)			.05			4.250 (1, 63)			.04	
		IIG	27.55 (3.65)		N/A		28.91 (3.75)													
		WLG	28.88 (4.16)		N/A		28.91 (3.97)													
**BFNE^d^**											1.065 (1, 62)			.14^c^			0.630 (1, 62)			.01^c^
	IIG		27.91 (7.89)		N/A		27.76 (6.74)													
	WLG		29.32 (4.92)		N/A		30.48 (5.27)													
**SADS^e^**											0.730 (1, 58)			.77^c^			4.173 (1, 58)			.32^c^
	IIG		84.13 (19.72)		N/A		81.35 (20.48)													
	WLG		86.21 (15.39)		N/A		85.07 (16.60)													
**TAS^f^**											0.065 (1, 61)			.60^c^			1.423 (1, 61)			.67^c^
	IIG		46.50 (9.63)		N/A		45.16 (11.01)													
	WLG		45.68 (10.34)		N/A		44.81 (9.25)													
**STAI-S^g^**											0.427 (1, 62)			.61^c^			0.054 (1, 62)			0.71^c^
	IIG		39.00 (8.75)		N/A		38.41 (9.85)													
	WLG		40.84 (10.94)		N/A		41.13 (9.99)													
**STAI-T^h^**											2.514 (1, 59)			.83^c^			5.299 (1, 59)			.18^c^
	IIG		41.80 (10.16)		N/A		39.00 (11.18)													
	WLG		42.03 (10.19)		N/A		41.52 (10.93)													
**CESD^i^**											0.370 (1, 61)			.82^c^			5.617 (1, 61)			.15^c^
	IIG		9.69 (9.88)		N/A		6.91 (6.99)													
	WLG		10.55 (9.01)		N/A		8.90 (9.89)													
**KRQ^j^**											3.804 (2, 106)			.17^k^			0.381 (2, 106)			.62^k^
	IIG		178.58 (27.56)		184.50 (28.23)		183.50 (31.97)													
	WLG		184.00 (23.52)		182.90 (23.65)		183.00 (24.61)													
**GES^l^**											0.231 (2, 110)			.38^k^			0.251 (2, 110)			.45^k^
	IIG		80.10 (13.61)		79.03 (20.34)		80.48 (19.03)													
	WLG		82.39 (13.64)		81.68 (13.39)		81.46 (13.41)													
**SWBS^m^**											0.307 (2, 114)			.21^k^			0.432 (2, 114)			.95^k^
	IIG		67.04 (16.00)		67.39 (18.19)		68.46 (18.14)													
	WLG		64.45 (16.46)		65.29 (15.14)		64.84 (14.24)													

^a^WHOQOL-BREF: World Health Organization’s Quality of Life–Brief Version.

^b^N/A: not applicable.

^c^Bonferroni multiple corrections (*P* value×7) for a total of 7 psychological scales (World Health Organization’s Quality of Life–BREF, Brief Fear of Negative Evaluation Scale, Social Avoidance and Distress Scale, Toronto Alexithymia Scale, State-Trait Anxiety Inventory–State, Sate-Trait Anxiety Inventory–Trait, and Center for Epidemiologic Studies Depression Scale).

^d^BFNE: Brief Fear of Negative Evaluation Scale.

^e^SADS: Social Avoidance and Distress Scale.

^f^TAS: Toronto Alexithymia Scale.

^g^STAI-S: State-Trait Anxiety Inventory–State.

^h^STAI-T: State-Trait Anxiety Inventory–Trait.

^i^CESD: Center for Epidemiologic Studies Depression Scale.

^j^KRQ: Korean Resilience Quotient.

^k^Bonferroni multiple corrections (*P* value×3) for a total of 3 psychological scales (Korean Resilience Quotient, General Self-Efficacy Scale, and Spiritual Well-Being Scale).

^l^GES: General Self-Efficacy Scale.

^m^SWBS: Spiritual Well-Being Scale.

### Impact of the Game on Nature Relatedness, Nutrition Status, and Psychological State Across the Entire Sample

As both IIG and WLG ultimately played the game, we performed additional analyses on the changes in each scale from before to after the game for the entire sample of these 2 groups combined. Multimedia Appendices 5 and 6 present the overall changes in nature relatedness, nutritional status, and psychological state for all participants before and after gameplay. There were statistically significant increases in the total NR score (*t*_62_=−4.150; *P*<.001) and total NQ score (*F*_2,114_=4.775; *P*=.01) after the 3-week game period. The total WHOQOL-BREF score also significantly increased after gameplay (*t*_63_=−4.027; *P*<.007). The STAI–Trait score (*t*_59_=3.765; *P*<.007) statistically significantly decreased following gameplay compared with baseline. No statistically significant differences were found for any of the other psychological scales (all *P*>.05).

## Discussion

### Principal Findings

Our study explored the impacts of a nature-friendly, rice-farming action video game on urban dwellers during the social distancing period of the COVID-19 pandemic, specifically examining changes in nature relatedness, nutritional behavior, and psychological state [37] The digital game intervention used in this study, similar to the concept of drug repositioning or the discovery of therapeutic factors from existing substances, sought to repurpose a recreational activity to take advantage of its therapeutic benefits [38]. This approach is gaining importance in the age of digital therapies and the emerging metaverse, where synergistic interactions between virtual and real-world experiences can potentially enhance mental health, promote healthy nutrition, and foster a deeper connection with nature [39,40].

As hypothesized, the results revealed significant changes after 3 weeks of gameplay in the total NR score and the *self* subscale of the NR in the IIG compared with the WLG. This indicates that the game intervention bolstered participants’ connection with nature, especially by influencing their personal relationships. For urban dwellers, who are increasingly disconnected from nature, this indicates a promising strategy for nurturing an affinity with the natural environment [5]. The enhancement in nature relatedness could have clinical applications, particularly in the context of eco-friendly therapeutic approaches, which are emerging as effective components of mental health treatment [41]. By fostering a connection with nature, such game interventions may provide a complementary tool for treating affective disorders and stress-related conditions. They can also be incorporated into mindfulness-based therapies to augment the therapeutic experience, especially for those with limited access to natural settings [42]. These implications suggest a valuable role for nature-centric games in the broader spectrum of mental health care approaches.

These findings are particularly relevant in light of the ongoing COVID-19 pandemic, which has necessitated social distancing and led to an increase in indoor activities, such as video gaming [17]. Video games, accessible through various platforms, including computers, consoles, and mobile devices, have been recommended by the World Health Organization as a means of alleviating stress [43,44]. In this context, our results suggest that video games have the potential to enhance nature connectivity, which is encouraging. This opens up possibilities for the development of the so-called serious games designed with therapeutic objectives [43].

The study also found significant changes in the total WHOQOL-BREF scores in the IIG compared with the WLG, indicating potential positive impacts on psychological state. These findings suggest that nature-friendly video games have an impact on the quality of life in general, in addition to improving specific psychological and nutritional aspects [38]. However, it was not possible to conclusively attribute these changes to specific factors in the video game, such as the joy derived from the gameplay or the cathartic impact of the narrative of the game.

The observed improvements in the WHOQOL-BREF scores may have therapeutic implications for individuals with diminished quality of life due to chronic conditions or individuals undergoing rehabilitation [45]. Aspects of the game that enhance psychological well-being could be particularly beneficial in clinical settings, offering a noninvasive means to support traditional therapies. Such games could be explored as a method to deliver positive psychological interventions, potentially aiding in the management of conditions such as chronic pain or fatigue, where patient engagement in enjoyable activities has been shown to contribute to the overall well-being.

For additional analysis, we investigated the pregame and postgame results for all participants. The findings showed significant changes in the total NR score, scores of the *self* and *experience* subscales of the NR, the total NQ score, and score of the dietary behavior subscale. In psychological terms, not only did the total WHOQOL-BREF score and scores of all of its subscales show significant changes, but the Social Avoidance and Distress Scale, Toronto Alexithymia Scale, STAI, and Center for Epidemiologic Studies Depression Scale Revised scores also showed significant changes. Although the pre-post comparison of all participants was limited to the same group without comparison, the broad positive changes in nature relatedness, nutritional status, and psychological states observed when the sample size was increased can be considered a basis for extrapolating the therapeutic and educational effects of a game intervention with targeted content and features. If future studies can ensure sufficient group-specific sample sizes, we can expect these findings to be further emphasized. In addition, the observation of dietary behavior changes through a nature-friendly serious game in this study raises the possibility of using such games as part of digital therapies for improving and managing dietary habits among urban dwellers.

Our study used the commercial game *Sakuna: Of Rice and Ruin*, which was launched by the South Korean branch of Nintendo in November 2020. The participants were randomized into the IIG and WLG conditions to evaluate the effects of the video game intervention. From the protective effects of nature on mental health [46], it can be assumed that an increased connection with nature through gaming may have some association with improved mental health and quality of life. Although our study showed significant results in terms of the NR, a case-control study examining the impact of specific game elements on nature connectivity could offer more precise insights. Furthermore, well-designed studies are necessary to ascertain which game factors facilitated the observed changes in the NR.

Previous studies have presented mixed results, suggesting that future research should prioritize understanding the long-term effects of gamification and the elements contributing to its efficacy [21]. It is crucial to identify the *active ingredients* that drive sustained behavioral changes and how they interact with individual differences, including personality traits and demographics [47]. Moreover, comprehensive longitudinal studies are essential to investigate gamification's long-term impact [20]. Although gamification shows promise in enhancing health and educational outcomes, its long-term effectiveness and core components require further exploration.

### Strengths and Limitations

This study has several strengths. First, it uses a commercial game for therapeutic and educational purposes. Using *Sakuna: Of Rice and Ruin*, we offered a real-world context for the exploration of the potential impact of video games on nature connectivity, dietary behavior, and psychological states. Similar to exploring the development of new drugs using natural plants or existing drugs, exploring therapeutic or educational elements in existing commercial games will be necessary in the coming digital age. This study has significant strengths in that regard. Second, the study design, which incorporated the random allocation of participants into either the IIG or the WLG, helped reduce the risk of selection bias, thereby improving the reliability of the findings. Third, the comprehensive evaluation of a broad range of outcomes, including changes in nature relatedness, nutritional status, quality of life, and various psychological measures, provided a multifaceted perspective on the potential benefits of video game interventions.

This study has some limitations. First, although the period in which we conducted our study was unique, with the COVID-19 pandemic spreading in South Korea and social distancing being mandated as a national policy, this study primarily targeted the general public, and the participants were not a patient group. Most psychological scales used in this study were designed for psychological and pathological conditions; because we targeted a general population, and not a clinical group, the baseline values of the psychological scale were not high. This posed limitations for the evaluation of the game’s psychological improvement effects compared with a patient group. Future studies would benefit from examining the effects on patient groups with psychological or physical issues or high-risk groups. Second, this study did not clearly identify the specific elements of the video game that contributed to the observed behavioral and emotional changes. Further research is needed to determine which elements of the game are the most influential in producing significant effects. Finally, the sample size in this study was relatively small to determine statistical significance. To increase the reliability and statistical power of the findings, it is necessary to conduct another study with a larger sample size. Along these lines, it is also necessary to confirm the evidence produced through additional independent studies to ensure reproducibility.

### Conclusion

Our study provides empirical evidence to suggest that nature-based video games, specifically *Sakuna: Of Rice and Ruin*, may enhance nature relatedness and positively influence nutritional and psychological states in an urban population. In light of our findings and the growing popularity of video games, particularly in the context of an increased tendency to stay at home owing to the COVID-19 pandemic, serious consideration should be given to the integration of digital games into strategies for promoting healthy behaviors and mental well-being. This is especially relevant as we navigate the challenges of our increasingly urbanized and digitized society. In conclusion, our study suggests that digital games, specifically those with a nature-based theme, can have a positive impact on users’ connection with nature, nutritional habits, and psychological health. Further studies are needed to corroborate these findings, identify the various influences of specific game elements, and determine the long-term effectiveness of these games as a form of digital therapy. Furthermore, it is necessary to optimize these specific game tools and integrate them into a real-world ecosystem to maximize their therapeutic potential.

## Appendix

Multimedia Appendix 1 Scenes captured from the game Sakuna: Of Rice and Ruin (images used with permission from Edelweiss, the video game developers).

Multimedia Appendix 2 Summary of game characteristics.

Multimedia Appendix 3 Summary of game components.

Multimedia Appendix 4 Significant findings regarding the impact of the game in the immediate invention group and waitlist group.

Multimedia Appendix 5 Impact of the computer game on nature relatedness, nutritional status, and psychological state in the entire sample.

Multimedia Appendix 6 Significant findings regarding the impact of the game in the entire sample.

Multimedia Appendix 7 CONSORT-eHEALTH checklist (V 1.6.1).

## Abbreviations

CRIS: Clinical Research Information Service
IIG: immediate intervention group
NQ: Nutrition Quotient Scale
NR: Nature Relatedness Scale
STAI: State-Trait Anxiety Inventory
WHOQOL-BREF: World Health Organization Quality of Life–Brief Version
WLG: waitlist group

## Data Availability

The data supporting the findings of this study are available upon request from the corresponding author. The data are not publicly available because of privacy concerns.
