# Supplementary material for: Effects of a Rice-Farming Simulation Video Game on Nature Relatedness, Nutritional Status, and Psychological State in Urban-Dwelling Adults During the COVID-19 Pandemic: Randomized Waitlist Controlled Trial
Source: J Med Internet Res. 2024 Jan 22;26:e51596. doi: 10.2196/51596 (PMC10845032; doi:10.2196/51596)
Supplement: Multimedia Appendix 1 [file jmir_v26i1e51596_app1.docx]

**Multimedia Appendix 1.** Captured scenes from the game "Sakuna: Of Rice and Ruin"

| **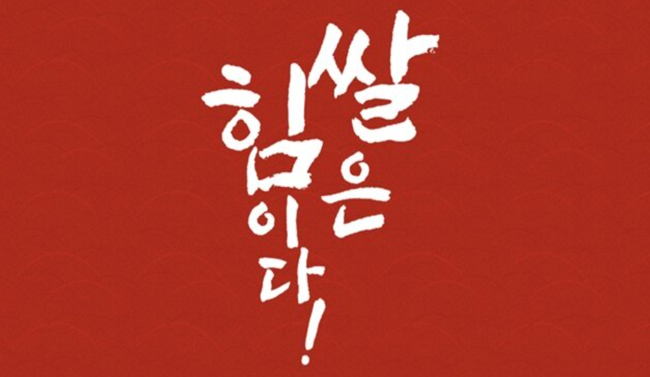**  **Opening Scene** | **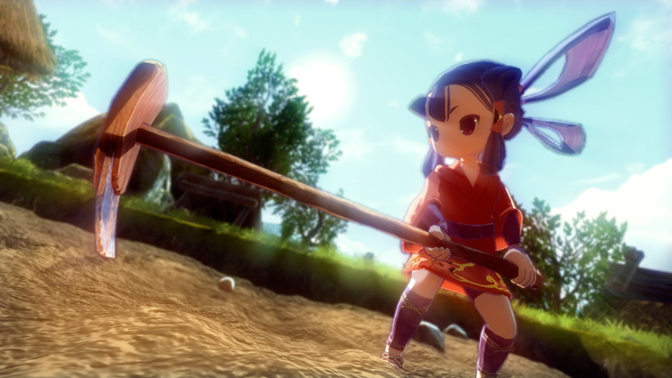**  **Grinding** |
| --- | --- |
| **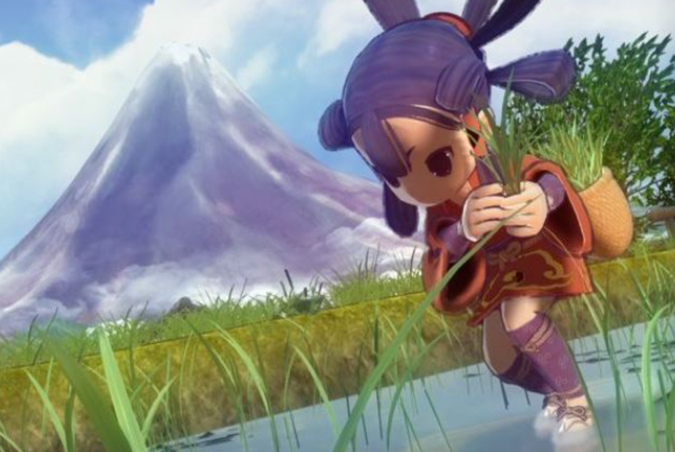**  **Transplanting rice 1** | **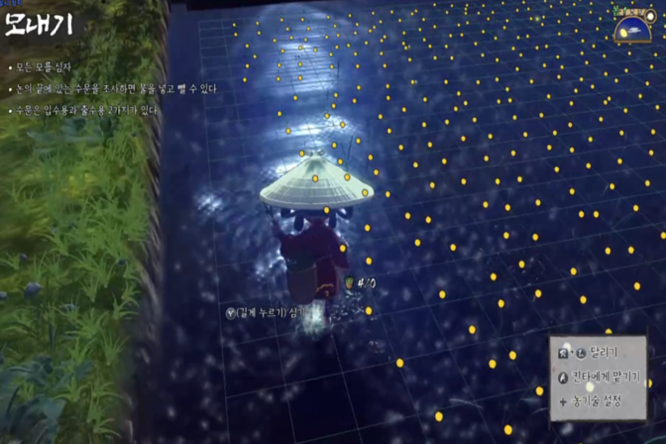**  **Transplanting rice 2** |
| **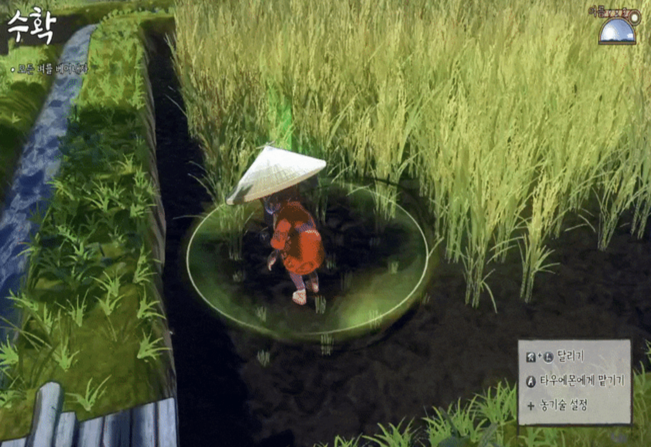**  **Harvest 1** | **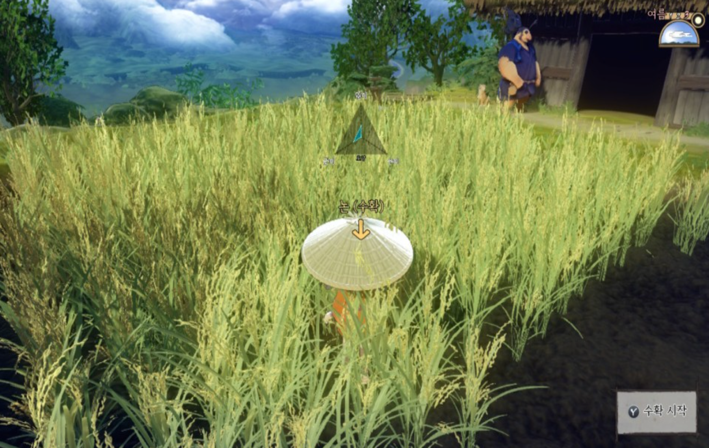**  **Harvest 2** |
| **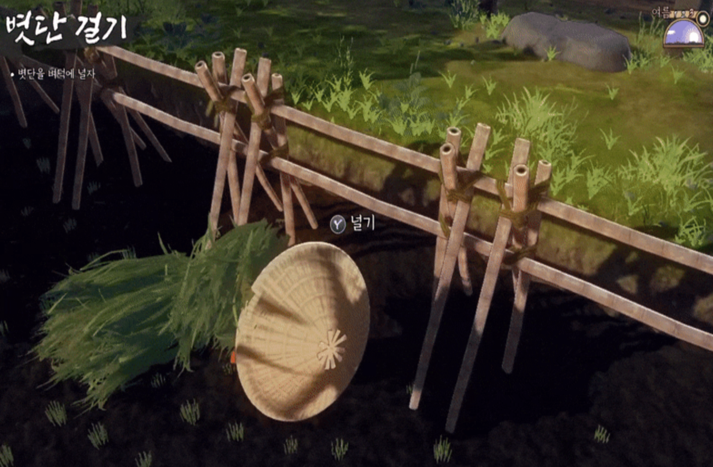**  **Hanging rice-sheaf** | **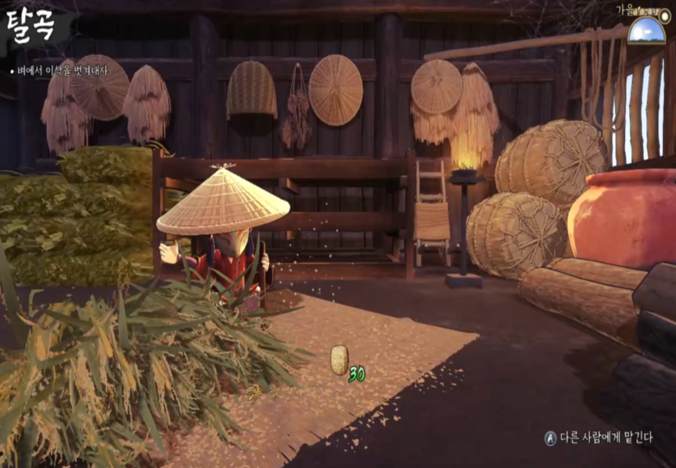**  **Threshing rice** |
| **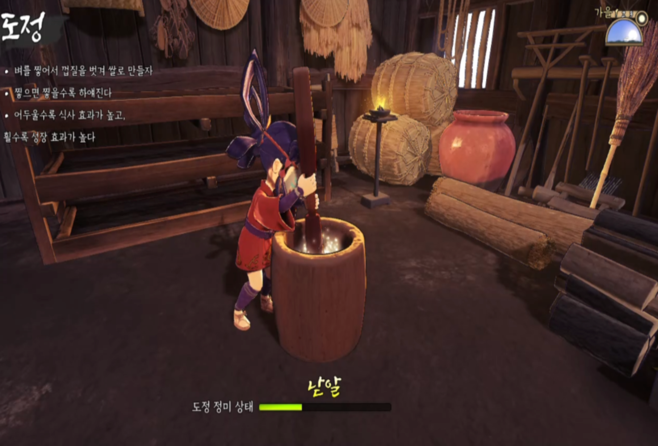**  **Milling rice scene** | **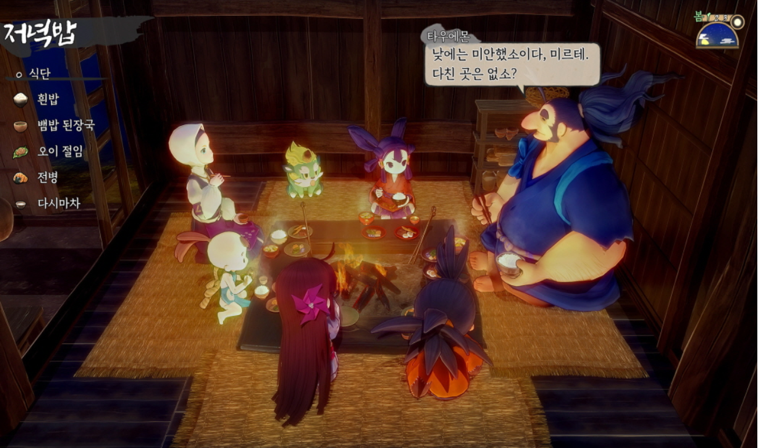**  **A scene of dinner with harvested rice and ingredients** |
| **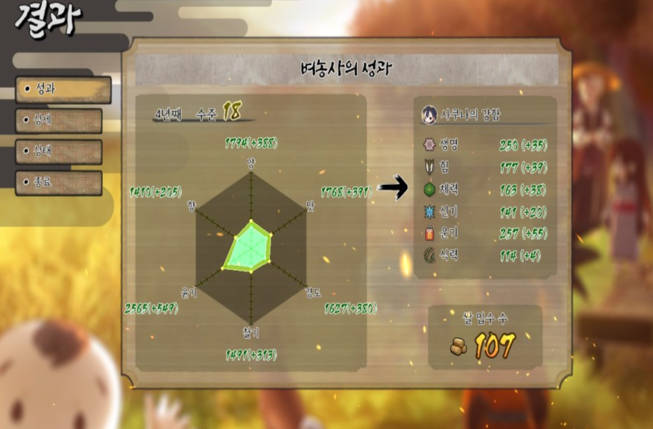**  **Showing the achivements of rice-farming** | **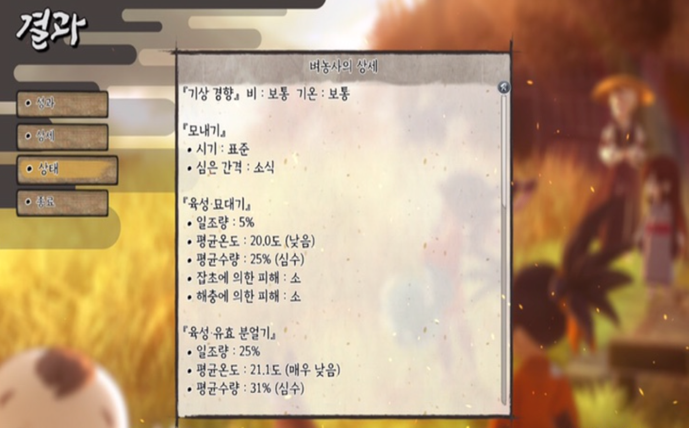**  **Showing the state of rice-farming** |
