# Supplementary material for: Effects of a Rice-Farming Simulation Video Game on Nature Relatedness, Nutritional Status, and Psychological State in Urban-Dwelling Adults During the COVID-19 Pandemic: Randomized Waitlist Controlled Trial
Source: J Med Internet Res. 2024 Jan 22;26:e51596. doi: 10.2196/51596 (PMC10845032; doi:10.2196/51596)
Supplement: Multimedia Appendix 2 [file jmir_v26i1e51596_app2.docx]

**Multimedia Appendix 2. Summary of Game Characteristics**

| **Basic characteristics** | |
| --- | --- |
| Genre | Action role-playing and simulation |
| Mode | Single-player |
| Release date | November, 2020 |
| Setting | Ancient Japan, rural place in an island |
| Topic | - Simulation of traditional manual rice-farming (individual and community labor with NPCs) and having family-dinner together with homemade food  - Others: Exploration of the environment, discover new areas for gathering, collect items necessary for survival, and clearing the demon monsters |
| Target player | Above 14 years (CERO: B rating) |
| Summary of the story | In a fictionalized ancient world, Princess Sakuna (the player’s role), the goddess responsible for the growth of grains, is cast down to an ancient Japanese island, where she needs to engage in rice-farming with outcast humans and produce good quality rice to regain her goddess status. Along with in-depth farming, she needs to clear the island’s monsters with farming tools as weapons and gather materials necessary for survival. The food choice she makes for dinner becomes her strength the next morning. By successful harvest of rice, collaboration with NPCs, and clearing of monsters, she can become true goddess |
| Rules | - Time: One cycle (day-night) is equivalent to a month. Three months consist of one season.  - Location: Outside the home region, there are 5 regions to explore, and one underground dungeon for the final quest.  - Feedback logs showing the association of rice scores and the player’s health status: Every time the player begins the game, a diary log appears with the health status on 6 sub-categories (life, strength, vitality, magic, luck, gusto=food power; on a score out of 100) and the current rice status level with a balance of 6 sub-categorical scores (yield, taste, hardness, stickiness, aesthetic, and aroma; as a hexagonal plot with full mark of 9999). The rice scores are directly linked to the player’s health status.  - Action rule: Weapon attacks with farming tools are available. There are four attack types (One-handed vs. Two-handed, combo- and special attacks). Damage is colored as blue (no damage due to high-defense of enemy), orange (good hit), red (weak enemy), and flash (“kill”, a critical hit or double damage). Maximum damage limit of 9,999,999, which cannot be exceeded.  - Death rule: If the player dies in an area, the progress so far is lost. Sakuna will reappear at the start location of that region of death. There is no penalty for death, but everything else is reset, including gathered items along the way. |
| Devices | PC (preferred), Nintendo Switch, PlayStation |
| Estimated play time | 35 hours |
