# Supplementary material for: Effects of a Rice-Farming Simulation Video Game on Nature Relatedness, Nutritional Status, and Psychological State in Urban-Dwelling Adults During the COVID-19 Pandemic: Randomized Waitlist Controlled Trial
Source: J Med Internet Res. 2024 Jan 22;26:e51596. doi: 10.2196/51596 (PMC10845032; doi:10.2196/51596)
Supplement: Multimedia Appendix 3 [file jmir_v26i1e51596_app3.docx]

**Multimedia Appendix 3. Summary of Game Components**

| Game components | |
| --- | --- |
| Agricultural component | - Simulation of in-depth traditional manual rice-farming (during four seasons: Winter/Spring/Summer/Autumn):  Land preparation (tilling the soil); Rice growing - Planting the seeds to waterbed, transplanting rice to the field, watering the field, 1^st^ tillering, 2^nd^ tillering, mid-summer drainage of water, adding fertilizer, use of ducks, spiders, snails, and frogs for reducing pests; Harvesting, threshing, and winnowing of rice.  - Farming records: Scores on the quality of growing rice (as a balance on the 6 aspects): 1) Yield, 2) Taste, 3) Hardness, 4) Stickiness, 5) Aesthetic, and 6) Aroma (→ These are directly reflected in power scales of Sakuna). |
| Psychological component | - Game graphics with surrounding nature and communities (NR; may reduce anxiety and depression): Resembling rural Asian scenes with rice paddies.  - Background music with natural sounds (NR; may reduce anxiety and depression) and traditional community-singing (may improve SA,  FNE, and alexithymia): Features the sounds of nature, such as peaceful night sounds of grasshoppers, ducks, frog croaks, and water streaming. Singing together the old Japanese rice-paddy folk songs when planting rice together.  - Friends/family and community support (may improve QoL, SA,  FNE, and alexithymia): Rice cultivating work, community conference, dinner making and sharing, exploration of the area, final year celebration.  - Interacting with animals that help in agriculture or exploration (NR; may improve anxiety, depression, QoL, SA,  FNE, and alexithymia): dogs (for exploration of regions together, up to 5 dogs available), cats (for petting, up to 5 cats available), mallard ducks (for eggs and weed removal in the rice field), and cow (for plowing the rice paddy together). |
| Action component | - Fighting and clearing of monsters with farming tools (side-scroll)  - Exploring nearby regions |
| Dietary component | - Making your own healthy daily dinner from available natural ingredients (either gathered, hunted, harvested, or stored): Promotion of well-balanced, lower-calorie homemade foods. Available sources include various grains, 10 kinds of meat, 11 kinds of seafood and freshwater fish, 18 kinds of vegetables and fruits, and 15 kinds of natural sauce and plants.  - Sharing daily meals with family and having dinner conversation with friends/family NPCs at home: Psycho-social influence on the player’s dietary behavior to be less “picky” and to eat a balanced meal.  - Expiration periods for the natural food ingredients and making of natural fertilizer with remnant food: Promotion of realistic and sustainable dietary behaviors.  - Eating a well-balanced meal increases Sakuna’s natural healing potential; missing a meal or major nutrients reduces the healing potential to zero, making it difficult to further explore other regions. |
| Available food components | |
| Available menu for dinner | - Rice (whole brown rice, half-polished rice, and white rice)  - Other grains (barley, Italian millet, and barnyard millet)  - Vegetables/fruits (18 kinds): beans, root vegetables, cucumbers..  - Natural sauce/spice plants(15 kinds): wasabi, green onions, garlic…  - Seafood/freshwater fish (11 kinds)  - Meats (10 different kinds) and edible insects (2 kinds)  - Various side dish: Soup (with 2 seafoods, 2 mushrooms, 2 kelps), bean-paste, soy-sauce, vinegar, oil (fish oil and plant oil), rice cakes, tofu, dried meats, pickled vegetables, sushi, haring, dried persimmon, pickled plum, taro, salmon, and rice-drinks |

NR: Nature-relatedness, QoL: Quality of life, SA: social avoidance, FNE: fear of negative evaluation
