# Supplementary material for: Effects of a Rice-Farming Simulation Video Game on Nature Relatedness, Nutritional Status, and Psychological State in Urban-Dwelling Adults During the COVID-19 Pandemic: Randomized Waitlist Controlled Trial
Source: J Med Internet Res. 2024 Jan 22;26:e51596. doi: 10.2196/51596 (PMC10845032; doi:10.2196/51596)
Supplement: Multimedia Appendix 4 [file jmir_v26i1e51596_app4.docx]

**Multimedia Appendix 4. Significant Findings of the Impact of Game between the Immediate Invention Group (IIG) and Waitlist Group (WLG).**


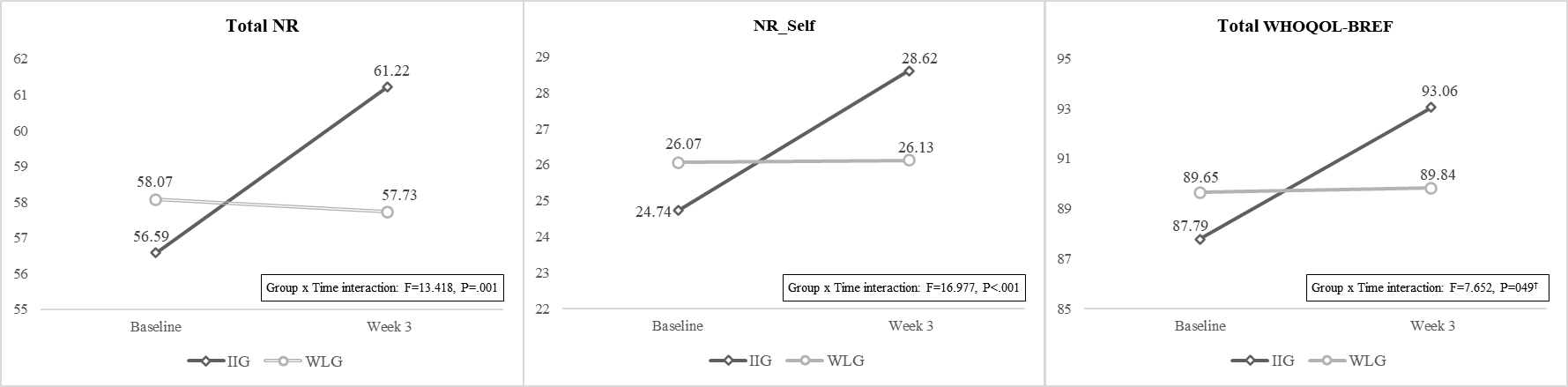


The results of repeated measures ANOVA revealed significant findings regarding Nature Relatedness (NR) and World Health Organization Quality of Life-BREF (WHOQOL-BREF) variables across time and groups.

†: Significance adjusted after Bonferroni correction.
