# Supplementary material for: Effects of a Rice-Farming Simulation Video Game on Nature Relatedness, Nutritional Status, and Psychological State in Urban-Dwelling Adults During the COVID-19 Pandemic: Randomized Waitlist Controlled Trial
Source: J Med Internet Res. 2024 Jan 22;26:e51596. doi: 10.2196/51596 (PMC10845032; doi:10.2196/51596)
Supplement: Multimedia Appendix 5 [file jmir_v26i1e51596_app5.docx]

**Multimedia Appendix 5. Impact of Computer Game on Nature-Relatedness, Nutritional Status, and Psychological State in Entire Sample**

| Measures | | Baseline, mean (SD) | 1 week after the start of playing the game, mean (SD) | 3 weeks after the start of playing the game, mean (SD) | *t* test (*df*) | *F* test (*df*) | *P* value |
| --- | --- | --- | --- | --- | --- | --- | --- |
| **NR^a^** | | | | | | | |
|  | Total NR | 57.35 (7.11) | N/A^b^ | 60.14 (6.86) | −4.150(*62*) |  | <.001 |
|  | Self | 25.49 (5.90) | N/A | 27.98 (5.60) | −4.866(*65*) |  | <.001 |
|  | Perspective | 15.86 (3.49) | N/A | 15.58 (3.21) | 1.047(*63*) |  | .23 |
|  | Experience | 15.59 (2.41) | N/A | 16.18 (1.99) | −2.532(*64*) |  | .01 |
| **NQ^c^** | | | | | | | |
|  | Total NQ | 49.56 (10.68) | 49.85 (9.73) | 51.67 (10.51) |  | 4.775(*2,114*) | .01 |
|  | Balance | 30.74 (17.05) | 28.30 (13.33) | 30.32 (14.59) |  | 2.286(*2,112*) | .11 |
|  | Diversity | 48.05 (16.82) | 50.93 (16.29) | 51.22 (16.23) |  | 2.916(*2,126*) | .06 |
|  | Moderation | 69.76 (12.94) | 69.37 (12.15) | 71.61 (13.27) |  | 2.494(*2,118*) | .09 |
|  | Dietary behavior | 44.72 (18.52) | 47.77 (16.72) | 50.18 (17.98) |  | 11.977(*2,124*) | <.001 |
| **WHOQOL-BREF^d^** | | | | | | | |
|  | Total score | 88.78 (12.02) | N/A | 92.67 (13.68) | −4.027(*63*) |  | <.007^e^ |
|  | Physical health | 24.32 (4.14) | N/A | 25.31 (4.74) | −2.746(*64*) |  | .008 |
|  | Psychological health | 19.61 (3.78) | N/A | 25.32 (4.70) | −12.688(*65*) |  | <.001 |
|  | Social relationships | 9.77 (2.04) | N/A | 10.35 (2.09) | −2.709(*64*) |  | .009 |
|  | Environmental health | 28.22 (3.84) | N/A | 29.05 (3.89) | −2.453(*64*) |  | .02 |
| BFNE^f^ | | 29.18 (6.77) | N/A | N/A | 0.986(*64*) |  | 2.30^e^ |
| SADS^g^ | | 84.58 (17.70) | N/A | N/A | 2.248(*59*) |  | .20^e^ |
| TAS^h^ | | 46.06 (9.85) | N/A | N/A | 2.035(*63*) |  | .32^e^ |
| STAI_S^i^ | | 40.06 (9.38) | N/A | N/A | 2.396(*63*) |  | .14^e^ |
| STAI_T^j^ | | 41.73 (10.54) | N/A | 39.20 (10.95) | 3.765(*59*) |  | <.007^e^ |
| CESD^k^ | | 9.19 (9.86) | N/A | 7.00 (8.36) | 2.427(*61*) |  | .13^e^ |
| KRQ^l^ | | 182.06 (26.28) | 184.96 (25.74) | 186.35 (29.51) |  | 3.237(*2,102*) | .13^m^ |
| GES^n^ | | 81.14 (13.41) | 80.50 (16.90) | 82.05 (16.71) |  | .811(*2,110*) | 1.28^m^ |
| SWBS^o^ | | 66.26 (15.03) | 66.63 (17.29) | 67.32 (16.69) |  | .547(*2,112*) | 4.06^m^ |

^a^NR: Nature Relatedness Scale.

^b^N/A: Not Applicable

^c^NQ: Nutrition Quotient Scale.

^d^WHOQOL-BREF: World Health Organization’s Quality of Life–BREF.

^e^Bonferroni multiple corrections (*P* value $\times$ 7) for a total of 7 psychological scales (World Health Organization’s Quality of Life–BREF [WHOQOL-BREF], Brief Fear of Negative Evaluation Scale [BFNE], Social Avoidance and Distress Scale [SADS], Toronto Alexithymia Scale [TAS], State-Trait Anxiety Inventory–State [STAI_S], Sate-Trait Anxiety Inventory–Trait [STAI_T], and Center for Epidemiologic Studies Depression Scale [CESD]).

^f^BFNE: Brief Fear of Negative Evaluation Scale.

^g^SADS: Social Avoidance and Distress Scale.

^h^TAS: Toronto Alexithymia Scale

^i^STAI_S: State-Trait Anxiety Inventory–State.

^j^STAI_T: Sate-Trait Anxiety Inventory–Trait.

^k^CESD: The Center for Epidemiologic Studies Depression Scale.

^l^KRQ: Korean Resilience Quotient.

^m^Bonferroni multiple corrections (*P* value $\times$ 3) for a total of 3 psychological scales (Korean Resilience Quotient [KRQ], General Self-Efficacy Scale [GES], and Spiritual Well-Being Scale [SWBS]).

^n^GES: General Self-Efficacy Scale.

^o^SWBS: Spiritual Well-Being Scale.
