# Supplementary material for: Effects of a Rice-Farming Simulation Video Game on Nature Relatedness, Nutritional Status, and Psychological State in Urban-Dwelling Adults During the COVID-19 Pandemic: Randomized Waitlist Controlled Trial
Source: J Med Internet Res. 2024 Jan 22;26:e51596. doi: 10.2196/51596 (PMC10845032; doi:10.2196/51596)
Supplement: Multimedia Appendix 6 [file jmir_v26i1e51596_app6.docx]

**Multimedia Appendix 6. Significant Findings of the Impact of Game in the Entire Sample.**

**
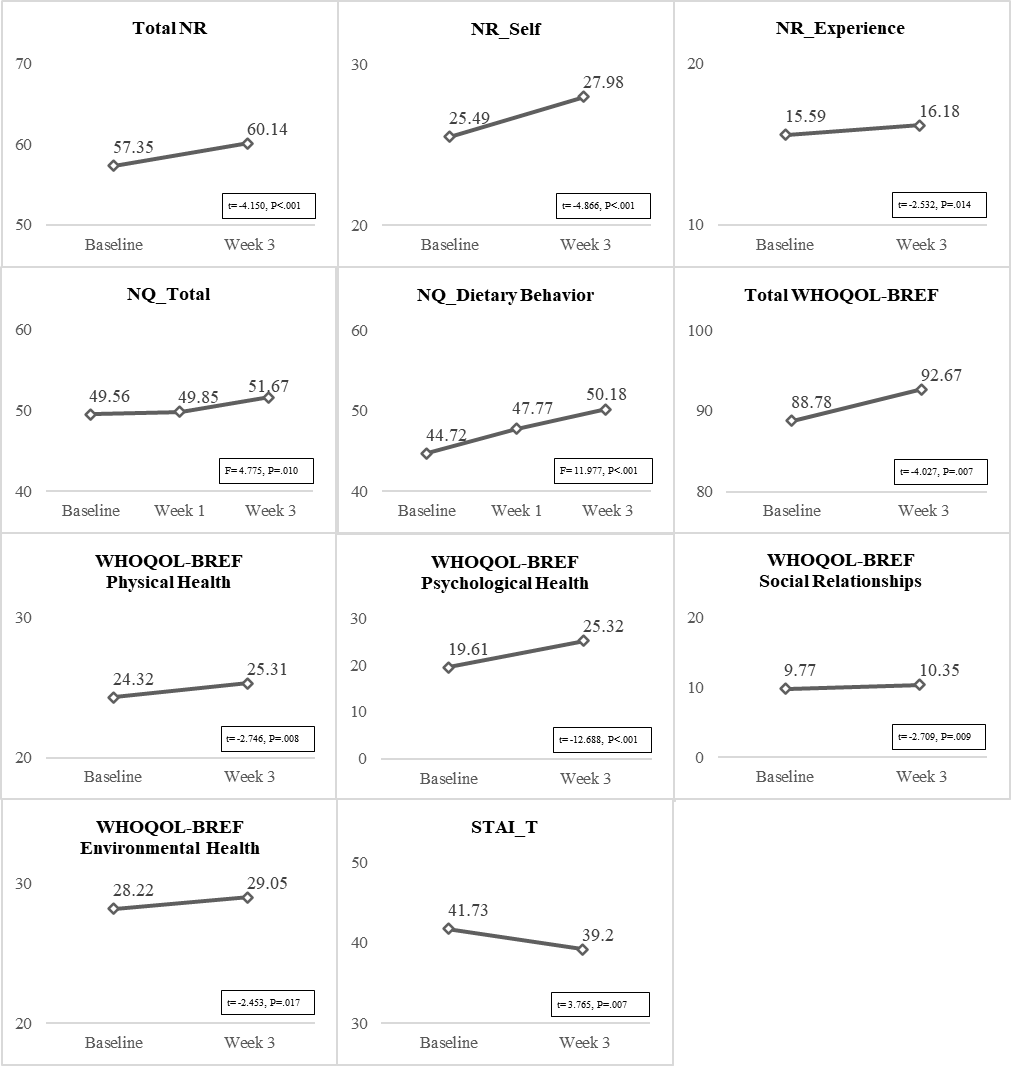
**

NR: The Nature Relatedness Scale; NQ: The Nutrition Quotient; WHOQOL-BREF: The World Health Organization Quality of Life-BREF; STAI_T: State-Trait Anxiety Inventory–Trait
